# An interrupted time series analysis of trends in opioid-related emergency department visits from pre-COVID-19 pandemic to pandemic, from the Canadian Hospitals Injury Reporting and Prevention Program

**DOI:** 10.1186/s12889-023-16414-z

**Published:** 2023-08-04

**Authors:** Xiaoquan Yao, Steven R. McFaull, André S. Champagne, Wendy Thompson

**Affiliations:** The Public Health Agency of Canada, 785 Carling Avenue, Ottawa, ON K1S 5H4 Canada

**Keywords:** Opioids, COVID-19, Interrupted time series analysis, Emergency department visits, Canadian Hospitals Injury Reporting and Prevention Program (CHIRPP), Fentanyl, Heroin

## Abstract

**Background:**

Opioid-related emergency department (ED) visits in Canada increased during the COVID-19 pandemic, but how trends in volume and case severity changed from pre-pandemic times through the pandemic is not known. Trends in ED visits related to specific types of opioids also remain unclear. Our objective was to describe pre-pandemic trends and how they changed with the onset of COVID-19 and thereafter.

**Methods:**

Based on data from the Canadian Hospitals Injury Reporting and Prevention Program, we identified opioid-related ED visits and constructed a time series from March 12, 2018 through March 7, 2021—two pre-COVID periods and one COVID period. We used an interrupted time series (ITS) analysis to examine trends in volume and case severity. We compared medians and means of monthly counts and percentages of severe cases between the periods, by sex, age, and opioid type.

**Results:**

Before the pandemic, there was an increasing trend in fentanyl-related visits for males, females and 25- to 64-year-olds, and a decreasing trend in heroin-related visits for males and 18- to 64-year-olds. Fentanyl-related visits for 18- to 24-year-olds showed an immediate increase at the start of the pandemic and a decreasing trend during the pandemic. Heroin-related visits for 12- to 17-year-olds had an immediate increase at the start of the pandemic; for 18- to 24-year-olds and 45- to 64-year-olds, the prior decreasing pre-pandemic trend ceased. For pooled opioid-related visits, no significant trend in the percentage of severe cases was observed throughout the entire study period.

**Conclusion:**

This study shows that an ITS approach in trend analysis is a valuable supplement to comparisons of before and after measures (with or without controlling seasonal effects). The findings provide evidence on how ED presentations for opioid use evolved in Canada from 2018 to 2021. The results can inform policies designed to reduce opioid-related harm in the context of a public health emergency.

**Supplementary Information:**

The online version contains supplementary material available at 10.1186/s12889-023-16414-z.

## Introduction

In Canada, the harms of opioid use increased during the COVID-19 pandemic [1, 2]. At the national level, there was a 91% increase in apparent opioid toxicity deaths and a 24% increase in opioid-related poisoning hospitalizations (Quebec not included) from April 2020 through March 2022, compared with the previous two years (April 2018 through March 2020) [1].

Several American studies have examined trends in opioid-related emergency department (ED) visits before and during the pandemic [3–8]. Canadian studies are scarce, but a table presenting opioid-related ED visits based on data from the National Ambulatory Care Reporting System (partial coverage of Canadian EDs) has been published [2, 9]. According to the table, the volume of opioid-related ED visits for selected months during the pandemic (March 2020 through June 2021), compared with the same months pre-pandemic (January 2019 through December 2019), initially decreased (March through May 2020) and then rose (June 2020 through June 2021). This type of comparison offers one perspective on pre-pandemic and pandemic contrasts, but it is not enough to understand the effect of pre-pandemic trends on the pandemic period and how trends changed with the onset of COVID-19 and thereafter.

To our knowledge, no Canadian studies have examined trends in ED visits related to specific types of opioids, such as fentanyl and heroin, before and during the pandemic. Wastewater surveys from five major Canadian cities, reflecting community consumption of opioids, suggested that trends for specific opioids differed: an increase in fentanyl, a decrease in codeine, and no significant change in morphine and methadone [10, 11]. Additionally, fentanyl and its analogues were reported more frequently in apparent opioid-related deaths and poisoning hospitalizations [1]. Trends in ED visits related to specific opioids were not clear.

As well, our literature search failed to find studies of trends in the severity of opioid-related ED visits before and during the pandemic. Public health restrictions and fear of contagion would likely affect health care-seeking behaviour, [12] resulting in only the more severe opioid-related cases going to the ED. Thus, we would expect the severity of ED visits to increase at the start of the pandemic.

The main objective of this analysis was to use an interrupted time series (ITS) approach [13] to examine trends in opioid-related ED visits from pre-pandemic through pandemic times. We aimed to describe the trend that existed before the pandemic and how it changed with the onset of COVID-19 and thereafter. We analysed trends in both volume and case severity for opioid-related ED visits. The second objective was to conduct sex-, age- and opioid type-specific analyses wherever sufficiently granular data were available.

## Methods

### Data source

Our study was based on ED visit data from the Canadian Hospitals Injury Reporting and Prevention Program (CHIRPP), an injury and poisoning sentinel surveillance system funded and administered by the Public Health Agency of Canada (PHAC) [14]. CHIRPP, which collects ED visit data, currently operates in 11 pediatric and nine general hospitals across Canada. At a visit to the ED of a CHIRPP-participating hospital, the patient or accompanying caregiver provides information about the time, place, and circumstances of the injury/poisoning event. The attending physician or other hospital staff then add clinical details, including substances involved, diagnosis (nature of injuries/poisonings, body parts affected), and treatment received. A data entry clerk at the hospital uses free-text variables to capture details of the event, including specific substances (if applicable) and information not coded by other variables. Coders at PHAC verify the data, perform further interpretive coding of free-text variables, and conduct data quality checks. Details about the development and uses of CHIRPP have been published elsewhere [15].

### Case selection for opioid-related injuries and poisonings

On March 23, 2022, we searched all CHIRPP cases entered on and before March 22, 2022. To identify opioid-related cases, we searched the text variables (i.e. narrative of injury event and substance) that provide information about substance use. For search terms, we consulted an online medication database [16]. The search terms (English and French) included opioids and related products—illicit drugs and prescription/over-the-counter medications containing opioids. To obtain the highest possible search sensitivity for medications, we included both generic and brand names, such as codeine, Tylenol #1 #2 #3 #4, oxycodone, OxyContin, etc. The medication used to treat opioid overdose, naloxone (Narcan), was also included in the search terms. We used SAS Perl regular expression [17] to identify synonyms, truncated terms, different spellings, and possible misspellings. The comprehensive search terms are available in the Appendix.

Identified cases were manually reviewed. Those in which opioids were not a factor in the poisoning/injury event (for example, taking opioid medication for pain relief after the injury event) were excluded. If a case reported only historic opioid use and the use was clearly related to the current ED visit, the case was included; otherwise, it was excluded. If naloxone was used to treat the overdose and had an effect on the patient, the case was included, even if no opioids were specified. If naloxone had no effect and no opioids were specified, the case was excluded. We enumerated each opioid reported. If an opioid was not specified, an “unspecified” category was assigned.

**Fig. 1:**
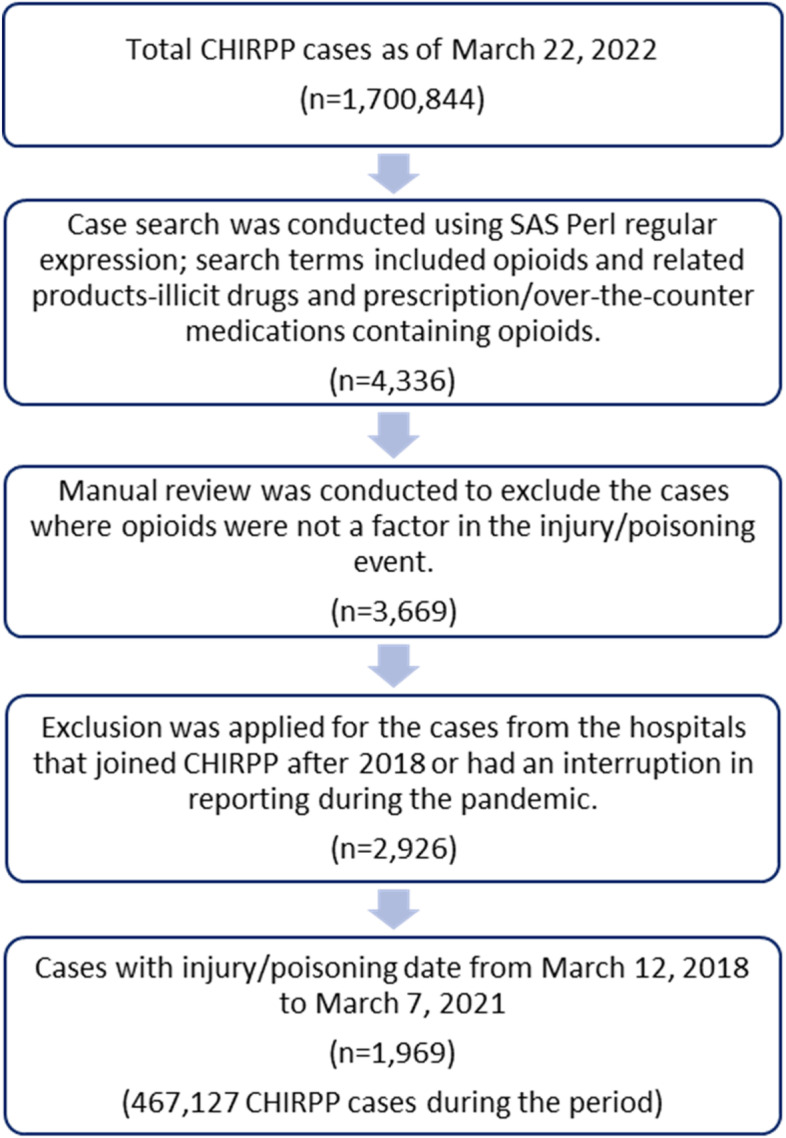
Case selection of opioid-related emergency department visits, CHIRPP, March 12, 2018 through March 7, 2021

CHIRPP was created in 1990 and is continuously expanding. Several hospitals joined the surveillance system after 2018 and some others experienced an interruption in reporting during the pandemic. We excluded those hospitals in the next step. As such, the opioid-related cases from 15 hospitals were included for this study, among which ten hospitals were pediatric. All these hospitals are located in urban areas. They span across the five regions of Canada: Atlantic Provinces, Central Canada, Prairie Provinces, West Coast and Northern territories. More specifically, the hospitals are located in the following provinces and territories: Newfoundland and Labrador, Nova Scotia, Quebec, Ontario, Manitoba, Alberta, British Columbia, and Northwest Territories. For the time series analysis, we included only cases with an injury/poisoning date from March 12, 2018 through March 7, 2021. Figure 1 illustrates the case selection process. The final count of cases was 1,969.

### Variables

The outcome variables were counts of opioid-related ED visits and the percentage that were severe. If a patient was admitted to the hospital because of the injury/poisoning event or died during the ED visit or at arrival, the case was considered severe. If the disposition of a case was unknown, the case was excluded from the severity analysis (*n* = 98). We checked the distribution of cases with unknown disposition by time and did not observe a pattern.

Demographic variables were biological sex and age. A gender analysis was not possible at the time. Age was grouped in years: younger than one, 1–4, 5–11, 12–17, 18–24, 25–44, 45–64, and 65 or older.

### Time series construction

The study period spanned three years: a two-year pre-pandemic window and a one-year pandemic window. Within a week from March 13, 2020, all provinces and territories had declared a public health emergency and implemented restrictions [18]. We defined the week including March 13, 2020 as the pandemic start week (March 9, 2020 through March 15, 2020). We examined 104 weeks before the start week (March 12, 2018 through March 8, 2020) and 52 weeks since the start week (March 9, 2020 through March 7, 2021). To ensure time unit uniformity, we defined a month as exactly four weeks. Three 13-month periods were analysed: pre-COVID-1 (March 12, 2018 through March 10, 2019); pre-COVID-2 (March 11, 2019 through March 8, 2020); and COVID (March 9, 2020 through March 7, 2021). Each period covered four seasons, thereby controlling for seasonal effects.

### Statistical analysis

We obtained monthly counts of pooled and specific opioid-related ED visits and calculated the percentage of severe cases in each month. An interrupted time series analysis (ITS) examined pre-pandemic and pandemic trends. We used the following regression model [13, 18, 19]:

$${\mathrm{Y}}_{\mathrm{t}} = {\upbeta }_{0} + {\upbeta }_{1}*{\mathrm{time}}_{\mathrm{t}} + {\upbeta }_{2}*{\mathrm{interruption}}_{\mathrm{t}} + {\upbeta }_{3}*{\mathrm{time\, after\, interruption}}_{\mathrm{t}} + {e}_{\mathrm{t}}$$

where Y_t_ is the outcome—monthly count or percentage of severe cases. β_0_ estimates the baseline level of the outcome; β_1_ represents the slope (existing trend) before the pandemic; β_2_ estimates the level change after the interruption (pandemic onset), indicating the immediate change at the interruption; and β_3_ is the slope change after the interruption. The sum of β1 and β3 represents the slope after the interruption [13, 19, 20]. The 27^th^ month (pandemic start month) is the interruption point. We used the generalised method of moments for coefficient estimation because of its robustness for non-linearity and heteroscedasticity [21, 22]. Seasonality was addressed by the balanced distribution of seasons before and after the interruption. In addition, we checked partial autocorrelation functions to get last significant lag and used the Newey-West autocorrelation adjusted standard errors [23] to address autocorrelation. We referenced a SAS macro [24] for the analysis.

We also calculated the median and mean of monthly counts and percentages of severe cases for each 13-month period (pre-COVID-1, pre-COVID-2, COVID) and the combined pre-COVID period. The purpose was to understand the effect of the trend on the summarized measures of each period. We used the Mann–Whitney U-test to compare monthly counts or percentages between periods or population groups. We also used a goodness-of-fit test to compare the total counts (annual counts) between periods.

We stratified analyses by sex, age and opioid type. We excluded cases with missing sex or age for sex- or age- specific analyses. For the age groups less than 1, 1–4, 5–11 and 65 + , the counts were 13, 88, 11 and 43, respectively, during the total 39 months at the pooled opioid level (the most majority of monthly counts were below five or even zero). We did not perform age-specific analyses for these age groups. For the visits involving the opioid types other than fentanyl and heroin (i.e. hydromorphone, oxycodone, codeine, morphine, methadone, tramadol), more than two-thirds of the monthly counts were lower than five at all sex and age level, so only annual counts were reported and compared between periods. For the severity analysis, if any month had an ED visit count below five, the percentage of severe cases for that month would be based on a small denominator, so we did not perform the analyse in case severity. Only pooled opioid-related visits were analysed—overall and for males, females, and 25- to 44-year-olds.

We defined statistical significance as *P* < 0.05 and used PC SAS 9.4 for the analysis [25].

## Results

### Characteristics of opioid-related cases

For the 15 hospitals included in this study, we identified 467,127 CHIRPP cases with an injury/poisoning date from March 12, 2018 through March 7, 2021; 1,969 were opioid-related. Table 1 compares the age, sex, and temporal distributions of opioid-related and all CHIRPP cases. More males than females were observed overall and among opioid-related cases. Patients in opioid-related cases tended to be older than those in all CHIRPP cases. The percentage of opioid-related cases among all CHIRPP cases was higher in the COVID period (March 9, 2020 through March 7, 2021), compared with the two pre-COVID periods (March 12, 2018 through March 10, 2019 and March 11, 2019 through March 8, 2020).

**Table 1:** Characteristics of opioid-related cases and all CHIRPP cases, March 12, 2018 through March 7, 2021

	**Opioid-related cases** N (column percent)	**All CHIRPP cases** N (column percent)	**N**_**opioid-related cases**_ **/** **N**_**all CHIRPP cases**_ _(X 1,000)_
**All**	1,969 (100%)	467,127 (100%)	4.2
**Sex**			
Male	1,186 (60.2%)	257,718 (55.2%)	4.6
Female	770 (39.1%)	206,554 (44.2%)	3.7
Not specified	13 (0.7%)	2,855 (0.5%)	4.6
**Age (min-25th-50th-75th-max)**^a^	0.0–17.9–31.5–42.5–96.3	0.0–5.2–12.0–19.4–107.1	Not applicable
**Age group**			
< 1	13 (0.7%)	17,181 (3.7%)	0.8
1–4	88 (4.5%)	96,565 (20.7%)	0.9
5–11	11 (0.6%)	120,064 (25.7%)	0.1
12–17	387 (19.7%)	112,217 (24.0%)	3.4
18–24	174 (8.8%)	18,766 (4.0%)	9.3
25–44	859 (43.6%)	43,844 (9.4%)	19.6
45–64	379 (19.2%)	32,505 (7.0%)	11.7
65 +	43 (2.2%)	25,548 (5.5%)	1.7
Unknown	15 (0.8%)	437 (0.1%)	34.3
**Injury period**			
Mar 12, 2018 – Mar 10, 2019	620 (31.5%)	167,555 (35.9%)	3.7
Mar 11, 2019 – Mar 8, 2020	566 (28.7%)	163,104 (34.9%)	3.5
Mar 9, 2020 – Mar 7, 2021	783 (39.8%)	136,468 (29.2%)	5.7

^a^Excludes cases with unknown age (the counts are noted under Age group in the table)

Among the opioids recorded in all opioid-related ED visits, fentanyl and heroin were the most common, followed by hydromorphone, oxycodone, codeine, morphine, methadone, and tramadol (Table 2).

**Table 2:** Interrupted time series analysis and comparison of CHIRPP opioid-related ED visits between pre-COVID and COVID

Sex/Age group	Variable	Coefficient estimates from interrupted time series analysis				Period				Statistical significance for period comparison			
						Pre-COVID			COVID				
		β_1_	β_2_	β_3_	β_1_ + β_3_	pre-COVID-1 (March 12, 2018—March 10, 2019)	pre-COVID-2 (March 11, 2019—March 8, 2020)	combined pre-COVID (March 12, 2018—March 8, 2020)	COVID (March 9, 2020—March 7, 2021)	pre-COVID-1 vs pre-COVID-2	pre-COVID-1 vs COVID	pre-COVID-2 vs COVID	combined pre-COVID vs COVID
**All sexes and age groups**													
	***Count***^a^												
	Pooled opioids					620	566	1186	783	-	*	*	*
	Fentanyl					148	237	385	456	*	*	*	*
	Heroin					167	107	274	95	*	*	-	*
	Hydromorphone					62	53	115	24	-	*	*	*
	Oxycodone					56	36	92	42	*	-	-	-
	Codeine					53	42	95	38	-	-	-	-
	Morphine					46	63	109	18	-	*	*	*
	Methadone					21	15	36	14	-	-	-	-
	Tramadol					11	12	23	12	-	-	-	-
	Unspecified opioids^b^					87	32	119	90	*	-	*	*
	***Monthly count***					**Median (Mean)**							
	Pooled opioids	0.038	1.560	1.758	1.797	46 (47.7)	41 (43.5)	44.5 (45.6)	55 (60.2)	-	*	*	*
	Fentanyl	0.705*	4.192	0.334	1.038	11 (11.4)	17 (18.2)	12.5 (14.8)	32 (35.1)	-	*	*	*
	Heroin	-0.330*	1.774	0.203	-0.126	12 (12.8)	7 (8.2)	10 (10.5)	7 (7.3)	*	*	-	*
	***Monthly proportion of severe cases***					**Median (Mean)**^**#**^				-	-	-	-
	Pooled opioids	0.001	-0.020	0.003	0.004	10.6% (12.1%)	14.7% (13.9%)	11.3% (13.0%)	15.7% (15.4%)	-	-	-	-
**Males**													
	***Monthly count***					**Median (Mean)**							
	Pooled opioids	0.025	2.953	0.837	0.863	29 (28.8)	23 (25.8)	28 (27.3)	35 (36.6)	-	-	*	*
	Fentanyl	0.439*	4.701	0.033	0.473	7 (8.1)	10 (11.7)	9 (9.9)	22 (23.4)	-	*	*	*
	Heroin	-0.256*	0.624	0.223	-0.033	8 (8.2)	< 5 (< 5)	6 (6.3)	< 5 (< 5)	*	*	-	*
	***Monthly proportion of severe cases***					**Median (Mean)**^**&**^							
	Pooled opioids	-0.000	0.021	0.004	0.003	7.0% (9.6%)	10.3% (10.9%)	10.3% (10.2%)	12.5% (14.1%)	-	-	-	-
**Females**													
	***Monthly count***					**Median (Mean)**							
	Pooled opioids	-0.023	-0.641	0.935	0.912	19 (18.8)	16 (17.0)	18.5 (17.9)	23 (23.4)	-	-	*	*
	Fentanyl	0.234*	0.120	0.327	0.560*	< 5 (< 5)	< 5 (5.8)	< 5 (< 5)	10 (11.5)	-	*	*	*
	Heroin	-0.074	1.150	-0.020	-0.093	< 5 (< 5)	5 (< 5)	< 5 (< 5)	< 5 (< 5)	-	-	-	-
	***Monthly proportion of severe cases***					**Median (Mean)**^**&**^							
	Pooled opioids	0.003	-0.081	0.003	0.006	13.3% (16.6%)	20.0% (19.2%)	17.1% (17.9%)	18.2% (17.2%)	-	-	-	-
**12–17**													
	***Monthly count***					**Median (Mean)**							
	Pooled opioids	0.176*	0.333	0.285	0.462*	7 (6.7)	9 (9.3)	8 (8.0)	14 (13.8)	-	*	*	*
	Fentanyl	0.033	1.124	0.181	0.214	< 5 (< 5)	< 5 (< 5)	< 5 (< 5)	< 5 (< 5)	-	*	*	*
	Heroin	0.019	2.372*	-0.124	-0.104	< 5 (< 5)	< 5 (< 5)	< 5 (< 5)	< 5 (< 5)	-	*	*	*
**18–24**													
	***Monthly count***					**Median (Mean)**							
	Pooled opioids	-0.161*	1.590	0.040	-0.121	6 (6.1)	< 5 (< 5)	< 5 (< 5)	< 5 (< 5)	*	*	-	-
	Fentanyl	0.026	1.766*	-0.222*	-0.196*	< 5 (< 5)	< 5 (< 5)	< 5 (< 5)	< 5 (< 5)	-	-	-	-
	Heroin	-0.118*	-0.009	0.150*	0.032	< 5 (< 5)	< 5 (< 5)	< 5 (< 5)	< 5 (< 5)	-	*	-	*
**25–44**													
	***Monthly count***					**Median (Mean)**							
	Pooled opioids	0.159	-1.761	0.775	0.934	20 (20.6)	18 (18.9)	18.5 (19.8)	23 (26.5)	-	-	-	*
	Fentanyl	0.436*	0.162	0.377	0.813	6 (6.2)	8 (10.2)	7 (8.2)	18 (19.5)	-	*	*	*
	Heroin	-0.128*	-0.744	0.029	-0.099	6 (6.2)	< 5 (< 5)	5 (5.0)	< 5 (< 5)	*	*	*	*
	***Monthly proportion of severe cases***					**Median (Mean)**^**#**^							
	Pooled opioids	0.001	-0.011	0.005	0.006	4.8% (4.5%)	7.1% (6.2%)	5.4% (5.3%)	7.1% (9.1%)	-	-	-	-
**45–64**													
	***Monthly count***					**Median (Mean)**							
	Pooled opioids	-0.062	-0.692	0.578*	0.516*	9 (9.8)	7 (8.2)	8 (9)	9 (11.2)	-	-	-	-
	Fentanyl	0.207*	0.107	0.084	0.291	< 5 (< 5)	< 5 (< 5)	< 5 (< 5)	7 (8.1)	-	*	*	*
	Heroin	-0.103*	0.137	0.136*	0.033	< 5 (< 5)	< 5 (< 5)	< 5 (< 5)	< 5 (< 5)	*	*	-	-

^a^Some cases had multiple opioids invloved, so the count of pooled opioid-related cases is less than the sum of opioid type-related cases

^b^Age and sex distribution by period for unspecified opioid-related cases: pre-COVID-1 [age: 12–17 (5.6%), 18–24 (18.0%), 25–44 (56.2%), 45–64 (16.9%); sex: male (73.0%), female (27.0%)]; pre-COVID-2 [age: 12–17 (18.2%), 18–24 (15.2%), 25–44 (36.4%), 45–64 (21.2%); sex: male (72.7%), female (24.2%), unspecified (3.0%)]; COVID [age: 12–17 (10.0%), 18–24 (7.8%), 25–44 (52.2%), 45–64 (23.3%); sex: male (64.4%), female (34.4%), unspecified (1.1%)]

^*^P < 0.05, -: *P* > 0.05

^#^*P* value for the differences between overall cases and 25- to 44-year-olds: < 0.05 for pre-COVID-1, pre-COVID-2 and combined pre-COVID; > 0.05 for COVID

^&^*P* value for the differences between males and females: < 0.05 for combined pre-COVID; > 0.05 for pre-COVID-1, pre-COVID-2, and COVID

### Volume trends

In terms of annual counts, there were more fentanyl-related ED visits and fewer heroin-, hydromorphone-, and morphine-related ED visits during COVID, compared with pre-COVID (Table 2).

**Fig. 2:**
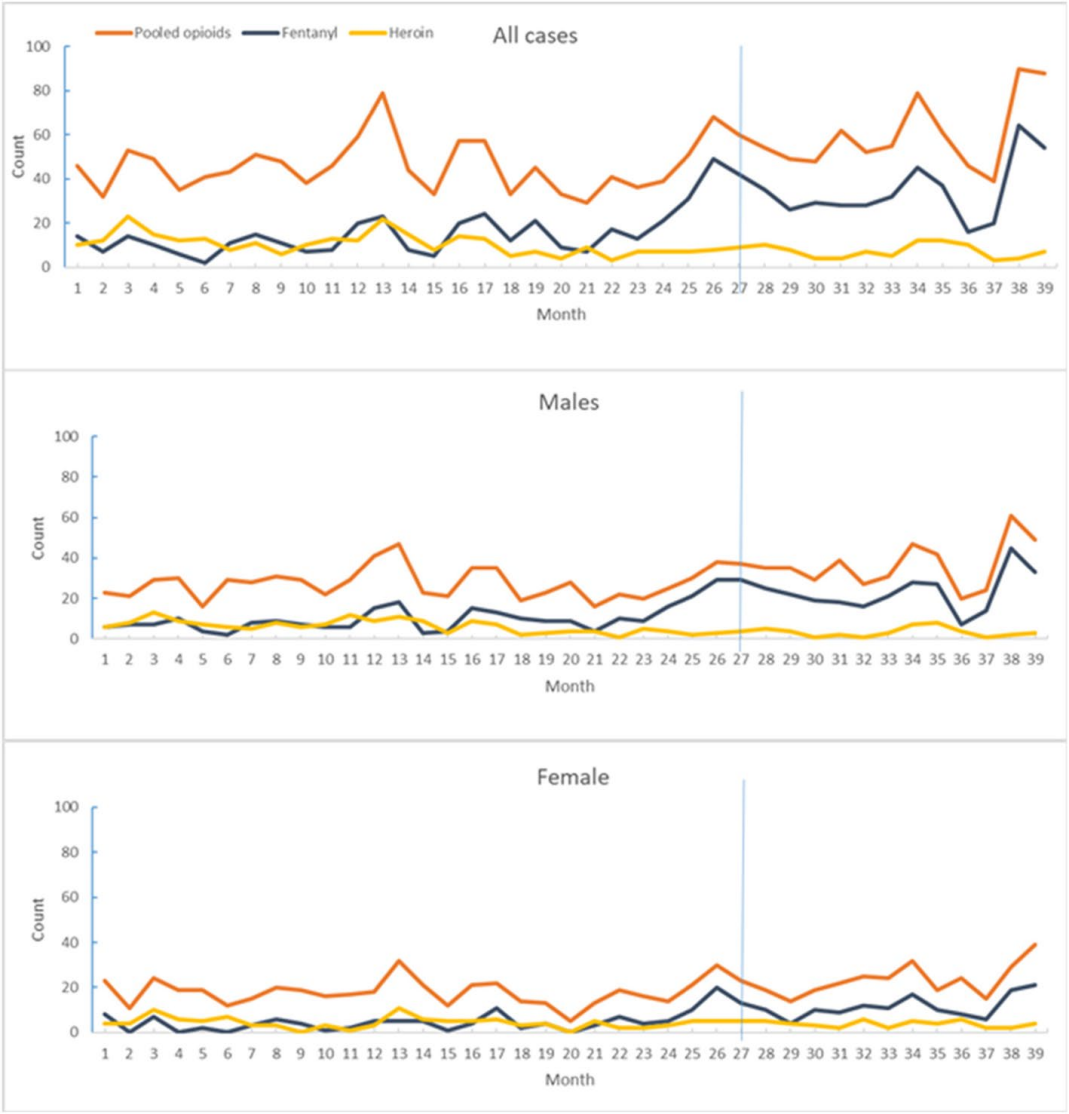
Counts of opioid-related emergency department visits, by sex, March 12, 2018 through March 7, 2021 Note: 1. Vertical blue line indicates the start of the pandemic. 2. See Appendix for the dates in the numbered months

**Fig. 3:**
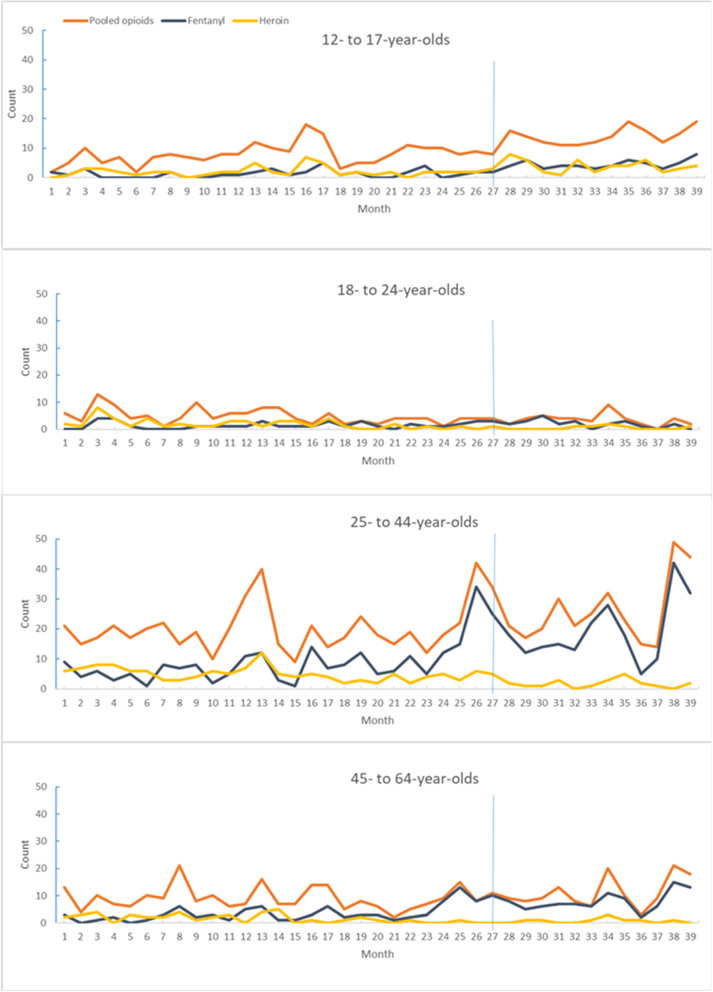
Counts of opioid-related emergency department visits, by age, March 12, 2018 through March 7, 2021 Note: 1. Vertical blue line indicates the start of the pandemic. 2. See Appendix for the dates in the numbered months

Figure 2 shows trends in monthly counts of ED visits related to pooled opioids, fentanyl, and heroin, overall and by sex. Figure 3 shows stratification by age group. Table 2 shows coefficient estimates from the ITS analysis and medians and means of monthly counts for the pre-COVID and COVID periods. The ITS analysis did not find a significant trend in ED visits related to pooled opioids for overall cases, males, or females. However, a significant increasing trend among 12- to 17-year-olds before the pandemic continued during the pandemic; a significant decreasing trend was observed among 18- to 24-year-olds before the pandemic; a significant change (increase in slope) after the interruption point was observed among 45- to 64-year-olds. Based on the medians and means of monthly counts, the volume of pooled opioid-related ED visits for overall cases increased significantly during COVID (median: 55/month; mean: 60.2/month), compared with the two pre-COVID periods combined (median: 44.5/month; mean: 45.6/month). A significant increase was apparent for males and females and for 12- to 17-year-olds and 25- to 44-year-olds.

For fentanyl-related ED visits, the ITS analysis showed a significant increasing trend before the pandemic with no level and slope change after the interruption point, overall or for males, females, and 25- to 64-year-olds. An increasing trend was observed among females after the interruption point. For 18- to 24-year-olds, an increase in level and a decrease in slope after the interruption point was observed. The median and mean of monthly counts for overall cases increased during COVID (median: 32/month; mean: 35.1/month), compared with pre-COVID (median: 12.5/month; mean: 14.8/month). A significant increase was also shown for males and females and for 12- to 17-year-olds and 25- to 64-year-olds.

For heroin-related ED visits, the ITS showed a significant decreasing trend before the pandemic, overall and for males and 18- to 64-year-olds. A significant increase in slope after the interruption point was observed for 18- to 24-year-olds and 45- to 64-year-olds; a significant level increase was evident for 12- to 17-year-olds. The median and mean of monthly counts were significantly lower during COVID (median: 7/month; mean: 7.3/month), compared with pre-COVID (median: 10/month; mean: 10.5/month), for overall cases. Males showed a significant decrease, but the change among females (decrease) was not significant. There was a significant increase for 12- to 17-year-olds, and a decrease for 18- to 44-year-olds.

### Case severity trends

**Fig. 4:**
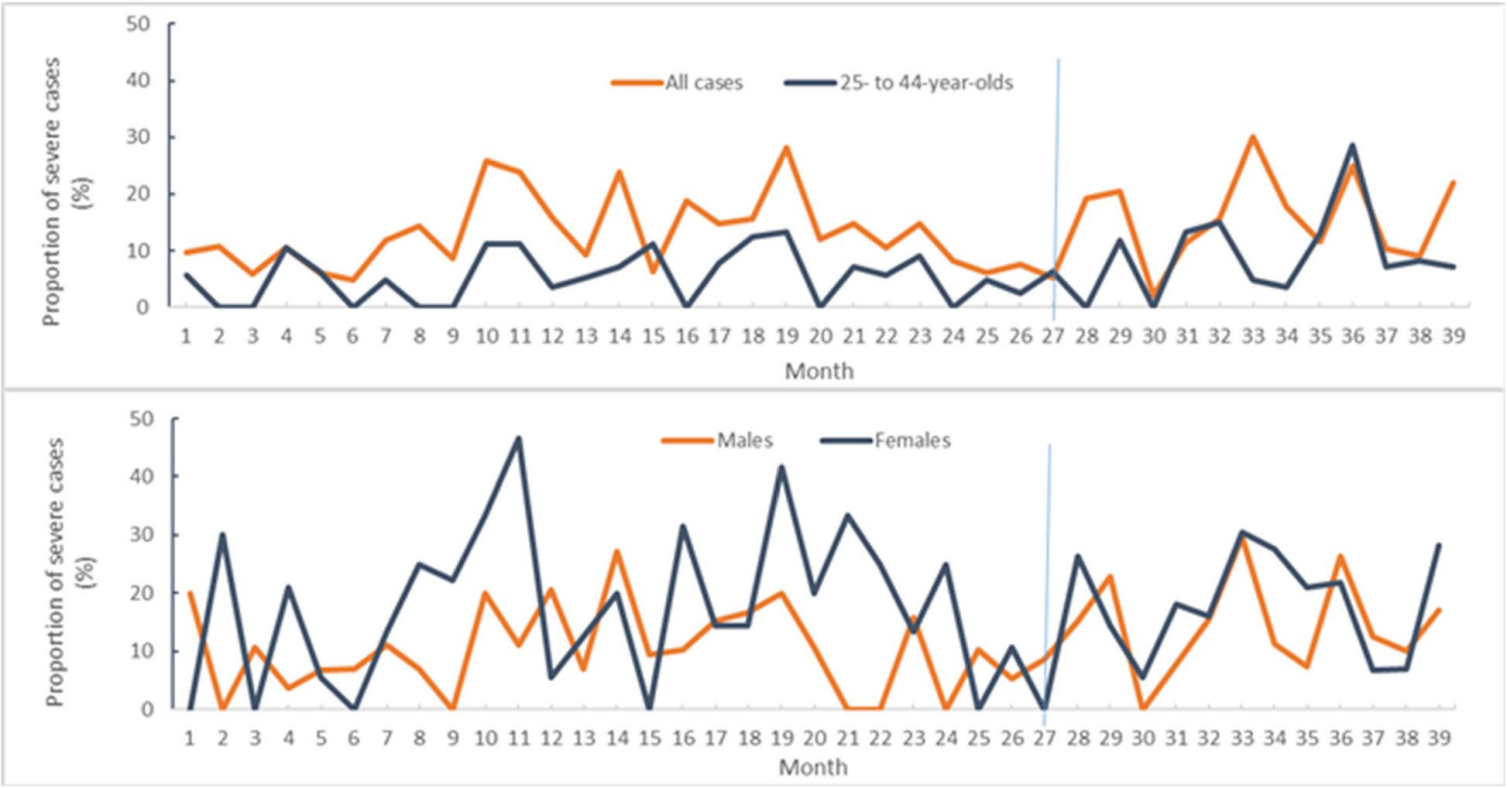
Percentage of severe cases among opioid-related emergency department visits, March 12, 2018 through March 7, 2021 Note: 1. Vertical blue line indicates the start of the pandemic. 2. See Appendix for the dates in the numbered months

Figure 4 shows the trend in the monthly percentage of severe cases among pooled opioid-related ED visits. The ITS analysis did not find any significant trend for the populations examined. Medians and means (Table 2) were all higher during COVID, compared with pre-COVID, overall and for males, females (except the mean), and 25- to 44-year-olds, even though the differences were not statistically significant. The percentage of severe cases was higher among females than among males for the combined pre-COVID period; the percentage was lower among 25- to 44-year-olds than among cases overall for any pre-COVID period.

## Discussion

Using data from CHIRPP, we examined trends in the volume and case severity of opioid-related ED visits from pre-COVID periods to the onset of the pandemic and the following year. Trends differed by sex, age, and opioid type. Before the pandemic, there was an increasing trend in fentanyl-related visits for males, females and 25- to 64-year-olds, and a decreasing trend in heroin-related visits for males and 18- to 64-year-olds. Fentanyl-related visits for 18- to 24-year-olds had an immediate increase at the start of the pandemic, followed by a decreasing trend during the pandemic. Heroin-related visits involving 12- to 17-year-olds also had an immediate increase at the start of the pandemic; for 18- to 24-year-olds and 45- to 64-year-olds, the pre-pandemic decreasing trend ceased because of a slope change during the pandemic. Throughout the entire study period, no significant trend in the percentage of severe cases was observed for pooled opioid-related ED visits.

Our main objective was to apply ITS to examine trends in opioid-related ED visits from pre-pandemic through pandemic periods to determine: What was the pre-pandemic trend? Was there an immediate change with the onset of COVID-19? How did the pre-existing trend change after pandemic onset? From a methodological point of view, our study supplements the method used in the table based on the National Ambulatory Care Reporting System [9] which calculated a percentage of change by comparing a number during the pandemic with another number during the same month before the pandemic, which will be thereafter referred as two-point (before and after) comparison method. Even though the seasonal effect was controlled in the method, another issue may arise. For example, our analysis shows an increasing pre-pandemic trend in the volume of fentanyl-related ED visits; that is, the volume was generally higher in the later pre-pandemic months than the earlier pre-pandemic months. Therefore, the later pandemic months were compared with a relatively higher volume (a greater denominator) than the earlier pandemic months. Therefore, if percentages were lower in later pandemic months than in earlier pandemic months, this would not necessarily mean a decreasing trend during the pandemic. This is not exactly the situation in the table [9], but we use it to illustrate the importance of considering a pre-existing trend when interpreting the percentages in the two-point comparison method. The ITS method considers the whole chain and offers information about the pre-existing slope, immediate change and slope change after an interruption. The ITS and two-point comparison methods each have their own value. They are complimentary when examining a trend.

Our study also compared medians and means of monthly counts between pre-pandemic and pandemic periods to understand the effect of the trend on these measures. For example, even though fentanyl-related ED visits did not show a significant increasing trend during the pandemic (there was substantial variability), the increasing pre-pandemic trend caused the level of fentanyl-related ED visits to be relatively high at the onset of the pandemic, and that level was maintained during the pandemic. Consequently, the median and mean were much higher during the pandemic, compared with the pre-pandemic periods.

Choosing an appropriate outcome variable is a consideration in an ITS analysis. One American study [5] used the percentage of opioid-related ED visits among all ED visits. This approach takes into account the overall reduction in ED visits, especially in the early phase of the pandemic. If ED visits due to all causes had been equally affected by pandemic circumstances such as public health restrictions and fear of contagion, the percentage would be an ideal measure. However, ED visits for some causes (for instance, motor vehicle collisions and sports-related incidents) [26, 27] may have been unusually low, which would reduce their weight in the denominator and cause the percentage of opioid-related visits to be inflated, even without a pandemic-related increase in substance use. Another American study [6] used the rate of opioid-related ED visits among the population as the outcome variable. If the population serviced by the CHIRPP sites was relatively stable during our study period, the count in our study is essentially the same as a rate.

In the context of public health, ITS studies have typically examined the effects of interventions, [19] as did a US study that assessed the impact of stay-at-home orders [6]. Although the time window was much shorter than in our study, and the results can be attributed to the stay-at-home order, the relatively short period could introduce seasonal bias. Our study, by contrast, covered a two-year pre-COVID period and one-year COVID period, balancing the seasonal effects before and after the interruption point. However, interpretation of the results would not be restricted to the effect of lockdowns, but rather, to a composite of many societal changes brought on by the pandemic. The immediate change at the pandemic onset could be explained by the lockdowns, such as the increase observed in fentanyl-related visits among 18- to 24-year-olds and heroin-related visits among 12- to 17-year-olds. During the one-year COVID period, the provinces and territories went through different schedules of lifting and reimplementing lockdown and other various measures adapting to the pandemic. As such, the trend found in this period cannot simply be attributable to the initial lockdowns. Our purpose was to use the principles of ITS to obtain insights into how the change during the pandemic was compared to the existing pre-pandemic trend. For example, among 25- to 64-year-olds, there was a clear increasing trend in fentanyl-related visits before the pandemic. The elevated level was maintained during the pandemic but did not become steeper. This may imply that the factors causing opioid-related harms prior to the pandemic, such as social and economic vulnerability, mental health, and contaminated illicit drug supply etc., still played the major role during the pandemic.

### Strengths and limitations

Our study shows the evolution of the trends in opioid-related ED visits from pre-pandemic through pandemic. Our analysis of ED visits related to specific opioids provides valuable insight. Pooled opioid-related ED visits did not show a significant trend in the ITS analysis. However, in the pre-pandemic period, fentanyl-related visits showed an increasing trend, while heroin-related visits were decreasing (thereby diluting the trend for pooled opioid-related visits). The sex- and age-specific analyses are also informative. The findings demonstrate the benefit of more granular analysis, compared with other studies, [3–9] particularly in support of tailored prevention and intervention efforts. For instance, we observed a decreasing trend starting before the pandemic in heroin-related ED visits among males, but not among females. We also observed an immediate increase at the start of the pandemic in fentanyl-related visits among 18- to 24-year-olds and heroin-related visits among 12- to 17-year-olds. The sex difference may reflect environmental, social, behavioural, or even biological differences by sex or gender among opioid users [28]. This highlights the importance of sex and gender considerations when implementing prevention and intervention measures. Our data show that adolescents and young adults were particularly impacted at the beginning of the pandemic, possibly reflecting the heavy toll of the lockdowns among this population. Adolescents and young adults are in a critical life stage for physical, social and emotional development. The disruption of daily life, such as closure of schools, sports, extracurricular activities, and other social venues, had a particular effect on this population. The resulted stress, social isolation, boredom, anxiety, and depression could have led to increased substance use [29, 30]. Also, the reduced substance use disorder treatment and supporting service could also have contributed to the immediate increase at the start of the pandemic [30]. This highlights the special considerations among this population in the future planning of emergency preparedness and management.

Our study is also unique in examining the trend in the percentage of severe cases among opioid-related ED visits. Public health restrictions and fear of contagion would likely affect health care-seeking behaviour, [12] and result in only the more severe opioid-related cases going to the ED. Thus, we would expect an increase in the severity of opioid-related ED visits at the start of the pandemic (level increase in ITS). However, we did not find a level or sustained increase among the populations examined. A possible explanation is that the CHIRPP hospitals are located in urban areas and underprivileged individuals may be overrepresented in our study cohort. During the pandemic, these individuals may not have had any other health care options available to them outside an ED (e.g., telehealth). Therefore, their use of an ED for non-severe cases may have remained the same.

Several limitations should be noted. First, because CHIRPP is a sentinel surveillance system, generalizability is restricted; rural and Indigenous populations are under-represented in CHIRPP. Second, most of the hospitals included in our study are pediatric, so adults were under-represented in the study, which affects the age distribution in Table 1. The age-specific analysis was intended to offset this limitation. Third, the small sample size limited our ability to analyse specific opioids and age/sex groups. Fourth, in our study, the opioid type among some cases could not be discerned. This could have impacted our opioid type-specific analysis. The pre-COVID-1 and COVID periods had more such cases than the pre-COVID-2 period, so pre-COVID-1 and COVID could have had more fentanyl or heroin cases. For the fentanyl-related visits, this means the observed increasing trend before the pandemic could have disappeared and the pandemic period could have presented an increasing trend. For the heroin-related visits, the decreasing trend before the pandemic could have been more pronounced. Most of these cases were males and 25–44-year-olds, for which the results could be the most potentially biased. Fifth, despite the robust data quality control measures in CHIRPP, errors may exist in data completeness and accuracy. Lastly, this study did not examine trends in the circumstances that surrounded opioid-related ED visits, which would provide useful information for prevention. Future work will investigate these factors.

## Conclusion

Our study used an innovative approach—an interrupted time series—to analyse trends in the volume and case severity of opioid-related ED visits from pre-COVID through COVID times. This method is a valuable supplement to comparisons of before and after measures (with or without controlling seasonal effects). The findings provide evidence on how emergency department presentations due to the harms of opioid use evolved in Canada from 2018 to 2021. The results can inform policy on reducing opioid-related harm in the context of a public health emergency.

### Supplementary Information

**Additional file 1. **

## Data Availability

The datasets used and analyzed during the current study are available from the corresponding author on reasonable request with privacy and confidentiality respected.

## Abbreviations

ED: Emergency department
ITS: Interrupted time series
CHIRPP: Canadian Hospitals Injury Reporting and Prevention Program
PHAC: Public Health Agency of Canada

## References

[1] Special Advisory Committee on the Epidemic of Opioid Overdoses (2022). Opioid- and Stimulant-related Harms in Canada (September 2022).

[2] Unintended consequences of COVID-19: Impact on harms caused by substance use, self-harm and accidental falls. Ottawa: Canadian Institute for Health Information. Available from: https://www.cihi.ca/en/covid-19-resources/impact-of-covid-19-on-canadas-health-care-systems/unintended-consequences. Accessed 15 Oct 2022.

[3] Holland KM, Jones C, Vivolo-Kantor AM, Idaikkadar N, Zwald M, Hoots B, Yard E, D'Inverno A, Swedo E, Chen MS, Petrosky E, Board A, Martinez P, Stone DM, Law R, Coletta MA, Adjemian J, Thomas C, Puddy RW, Peacock G, Dowling NF, Houry D (2021). Trends in US Emergency Department Visits for Mental Health, Overdose, and Violence Outcomes Before and During the COVID-19 Pandemic. JAMA Psychiat.

[4] Pines JM, Zocchi MS, Black BS, Carlson JN, Celedon P, Moghtaderi A, Venkat A (2021). How emergency department visits for substance use disorders have evolved during the early COVID-19 pandemic. J Subst Abuse Treat.

[5] Hall GT, Cruz DS, Lank PM, McCarthy DM, Kim HS (2021). Opioid-related Emergency Department Visits During COVID-19 in a Large Health System. J Addict Med.

[6] Root ED, Slavova S, LaRochelle M, Feaster DJ, Villani J, Defiore-Hyrmer J, El-Bassel N, Ergas R, Gelberg K, Jackson R, Manchester K, Parikh M, Rock P, Walsh SL (2021). The impact of the national stay-at-home order on emergency department visits for suspected opioid overdose during the first wave of the COVID-19 pandemic. Drug Alcohol Depend.

[7] Venkatesh AK, Janke AT, Kinsman J, Rothenberg C, Goyal P, Malicki C, D'Onofrio G, Taylor A, Hawk K (2022). Emergency department utilization for substance use disorders and mental health conditions during COVID-19. PLoS One.

[8] Soares WE, Melnick ER, Nath B, D'Onofrio G, Paek H, Skains RM, Walter LA, Casey MF, Napoli A, Hoppe JA, Jeffery MM (2022). Emergency Department Visits for Nonfatal Opioid Overdose During the COVID-19 Pandemic Across Six US Health Care Systems. Ann Emerg Med.

[9] Impact of COVID-19 on harms caused by substance use, March 2020 to June 2021 — Data Tables. Ottawa: Canadian Institute for Health Information. Available from: https://www.cihi.ca/sites/default/files/document/impact-covid-19-harms-caused-substance-use-march-2020-to-june-2021-data-tables-en.xlsx. Accessed 15 Oct 2022.

[10] Wastewater analysis suggests that consumption of fentanyl, cannabis and methamphetamine increased in the early pandemic period. Ottawa: Statistics Canada; 2021. Available from: https://www150.statcan.gc.ca/n1/daily-quotidien/210726/dq210726a-eng.htm. Accessed 15 Oct 2022.

[11] Wastewater analysis suggests that use of particular opioid pain medications decreased during the COVID-19 pandemic in 2020. Ottawa: Statistics Canada; 2022. Available from: https://www150.statcan.gc.ca/n1/daily-quotidien/220309/dq220309c-eng.htm. Accessed 15 Oct 2022.

[12] Pines JM (2020). COVID-19, Medicare for all, and the uncertain future of emergency medicine. Ann Emerg Med.

[13] Wagner AK, Soumerai SB, Zhang F, Ross-Degnan D (2002). Segmented regression analysis of interrupted time series studies in medication use research. J Clin Pharm Ther.

[14] Canadian Hospitals Injury Reporting and Prevention Program. Available from: https://www.canada.ca/en/public-health/services/injury-prevention/canadian-hospitals-injury-reporting-prevention-program.html. Accessed 15 Oct 2022.

[15] Crain J, McFaull S, Thompson W, Skinner R, Do MT, Fréchette M (2016). Status report. The Canadian Hospitals Injury Reporting and Prevention Program: a dynamic and innovative injury surveillance system. Health Promot Chronic Dis Prev Can.

[16] DrugBank online. Available from: https://go.drugbank.com/. Accessed 01 Apr 2022.

[17] Pattern Matching Using Perl Regular Expressions (PRX). Cary (NC): SAS Institute Inc. Available from : https://documentation.sas.com/doc/en/pgmsascdc/9.4_3.2/lefunctionsref/n13as9vjfj7aokn1syvfyrpaj7z5.htm. Accessed 15 Oct 2022.

[18] COVID-19 Intervention Timeline in Canada. Ottawa: Canadian Institute for Health Information; 2022. Available from: https://www.cihi.ca/en/covid-19-intervention-timeline-in-canada. Accessed 15 Oct 2022.

[19] Bernal JL, Cummins S, Gasparrini A (2017). Interrupted time series regression for the evaluation of public health interventions: a tutorial. Int J Epidemiol.

[20] Fusi F, Lecy J (2020). Interrupted time series.

[21] Hansen LP (1982). Large sample properties of generalized method of moments estimators. Econometrica.

[22] Evans RW (2018). Generalized Method of Moments (GMM) Estimation.

[23] Newey WK, West KD (1987). A simple, positive semi-definite, heteroskedasticity and autocorrelation consistent covariance matrix. Econometrica.

[24] Caswell J (2017). Interrupted time series analysis for single series and comparative designs: a guide for beginners with SAS macro.

[25] SAS Institute. SAS 9.4 Language Reference: Concepts (3rd. ed.). Cary (NC): SAS Institute Inc.; 2014. ISBN:978–1–62959–307–4.

[26] Rapoport MJ, Chee JN, Aljenabi N, Byrne PA, Naglie G, Ilari F (2021). Impact of COVID-19 on motor vehicle injuries and fatalities in older adults in Ontario. Canada. Acci Anal Prev.

[27] Keays G, Friedman D, Gagnon I (2020). Injuries in the time of COVID-19. Les blessures au temps de la COVID-19. Health Promot Chronic Dis Prev Can..

[28] McHugh RK (2020). The importance of studying sex and gender differences in opioid misuse. JAMA Netw Open.

[29] Lundahl LH, Cannoy C (2021). COVID-19 and Substance Use in Adolescents. Pediatr Clin North Am.

[30] Romero RA, Young SD (2022). Adolescents and opioid-related outcomes amidst the COVID-19 pandemic. J Addict Dis.

