# Supplementary material for: An interrupted time series analysis of trends in opioid-related emergency department visits from pre-COVID-19 pandemic to pandemic, from the Canadian Hospitals Injury Reporting and Prevention Program
Source: BMC Public Health. 2023 Aug 4;23:1483. doi: 10.1186/s12889-023-16414-z (PMC10401736; doi:10.1186/s12889-023-16414-z)
Supplement: Supplementary file 1 — Additional file 1. [file 12889_2023_16414_MOESM1_ESM.docx]

**Appendix**

1. SAS search terms for identifying opioid-related cases

pattern=prxparse('/

\s222[\s\.]|

\s282[\s\.]|

\s292[\s\.]|

[^t]ox[iy][ck]o|

[ck]odal|

[ée]torphine|

\sm\.?\s?o\.?\s?s\.?(\s|-sr)\s|

\sw(\s|\/|ith\s)cod|

a\.?\s?c\.?\s?(&|and)\s?c\.?\s|

ac[eé]morph|

ac[eé]t\s?[23]|

actacode|

airacof|

atasol|

avinza|

axacet|

axisal|

azocine|

b[eé]zitramide|

bion[io]ne|

bisoltus|

bolodorm|

bremazocine|

bromophar|

brompton|

bronchicum|

bronchodine|

bunavail|

buphine|

bupr[eé]norphine|

butorphanol|

c\.?\s?(&|and)\s?C\.?\s|

calmylin|

capCof|

cardanon|

co-?codamol|

co[nd]orfone|

co\s?-?dydramol|

coactifed|

cocet|

cod[eé]?in|

codal|

codant|

codar|

codedrill|

codeisan|

codenon|

codeprex|

coderpina|

codi[eé]ne|

codicalm|

codicept|

codinex|

codix\s?5|

codone|

codrix|

coducept|

contin\s|

cotabs|

cotridin|

cotrifed|

cougel|

\scoutan\s|

covan Syrup|

cyclazocine|

demerol|

dextromoramide|

dextrorphane?|

dex-tuss|

dezocine|

diacephine|

diaphorin|

diconal|

dihydro|

dim[eé]thylthiambutene|

dimetapp|

dinarkon|

dinco|

diph[eé]noxylate|

dolonovag|

dolophine|

drocode|

durela|

empracet|

emtec|

enadoline|

endaCof|

endal|

endocet|

endodan|

endone|

enk[ée]phaline?|

ethoheptazine|

etonitazene|

etoxeridine|

eubine|

euk?din|

eurodamine|

eutagen|

exalgo|

exdol|

exeClear|

expecto|

expectorant|

farmacod|

faxeladol|

fenta|phenta|

fentora|

fiorinal[\s-]C|

fur[eé]thidine|

galcodine|

ganituss|

gelonida|

gesic|

guaifenesin|

h[eé]ro[iï]n|

histex|

hydromo|

hydrocodone|

hydrocont|

hydrostat|

hysingla|

instanyl|

ionsys|

Iophen|

isalmadol|

isonip[eé]caine|

isotonitazene|

jurnista|

k[eé]tazocine|

k[eé]tob[eé]midone|

kadian |

ketogan|

kyotorphine?|

l[eé]f[eé]tamine|

l[eé]vorphanol|

lau?did|

lauda(co)?num|

lenoltec|

leptanal|

lexuss|

lop[eé]ramide|

lorcet|

lortab|

lortuss|

ludonal|

m-?eslon |

m[eé]p[eé]ridine|

m[eé]ridine|

m[eé]tazocine|

m[eé]thado|

m[eé]thadyle?|

m[eé]thylsamidorphan|

mar[\s-]cof|

maxi[\s-]Tuss|

m-clear|

medicodal|

m-end|

meptazinol|

mesehist|

metadol|

methoxacet|

methoxisal|

mor(ph|f)|

\sms contin\s|

ms[\s-\.]ir\s|

muscle\s?(&|and)\sback\spain\srel|

naloxo|

narcan|

narcobasin[ae]|

nargenol|

narodal|

nasotuss|

neo AC|

ninjacof|

norco|

norphine|

notuss|

novahistex|

novolaudon|

nsc\s?19043|

nu[\s-]acetaco|

nucodan|

nucynta|

olic[eé]ridine|

omnopon|

onsolis|

opiate|opioid|opium|

opton|

oradrine|

oripavine|

ossicodone|

oxanest|

oxecta|

\soxy\s|

\soxyco|

\soxy[\s\.]?ir|

\soxydose\s|

\soxyfast\s|

\soxygesic\s|

\soxykon\s|

\soxymorph\s|

\soxyneo\s|

\soxynorm\s|

p[eé]thidine|

pain relief|

painex|

pal[il]adon|

pancodine|

papaver|pavot|

parafon|

paregoric|

pavinal|

pecfent|

pectoral|

pentazocine|

pentuss|

perc[ao]cet|

percodan|

percolone|

ph[eé]nadoxone|

ph[eé]nazocine|

ph[eé]ncyclidine|

phenaphen|

phenaridine|

phenylhistine|

phrenilin|

pic[eé]nadol|

piminodine|

pipa[dn]one|

pluratuss|

pms[\s-]pharnal|

poly Hist|

pr[ao]cet|

pro[\s-]clear|

pro[\s-]red|

profadol|

promedol|

pronal|

pronarcin|

propiram|

propoxyph[eè]ne|

pseudodine|

ralivia|

ramadol|

rapinyl|

recuvyra|

relcof|

remoxy|

robafen|

robaxacet|

robaxisal|

robitussin|

rounox|

routec|

roxicet|

rydex|

samidorphan|

sedapain|

semcox|

sinthiodal|

sinutab|

\sSoma\s|

sophidone|

spasmhalt|

statex|

statuss|

stupenal|

suboxone|

subutex|

supeudol|

talwin|

tamicode|

tanyl|

tapentadol|

targin|

tebodal|

tecnal|

tekodin|

terpin|

teva[\s-]cotridin|

th[eé]ba[iï]ne|

thecodin|

tifluadom|

tilidine|

tl[\s-]hist|

tonazocine|

tramacet|

tramadol|

tramide|

triacin|

trianal|

triatec|

tricode|

tridural|

tusnel|

tussaminic|

tussin|

tussoret|

tuxarin|

tuzistra|

tylenol\s?#\s?[1-4]\s|

tylenol\s?[1-4]\s[^(tablet)]|

tylenol\sno\.?\s?[1-4]\s|

tylenol\sw(\s|\/|ith\s)cod|

U-47700|

ultram|

vic[ao]din|

ampole|

wildnil|

z[\s-]tuss|

zodryl|

zohydro|

zotex|

zubsolv|

zytram|

etazene|

brorphine|

metodesnitazene|

nortilidine|

metonitazene|

carbonyl[\s-]bromadol|

ap[\s-]238|

o[\s-]AMKD|

isotonitazene|

2[\s-]methyl[\s-]AP[\s-]237|

tianeptine|

piperidylthiambutene|

protonitazene|

2f[\s-]viminol

/i');

1. Dates in the months of the time series

| Month | Dates | Month | Dates | Month | Dates |
| --- | --- | --- | --- | --- | --- |
| 1 | 12Mar2018 – 08Apr2018 | 2 | 09Apr2018 – 06May2018 | 3 | 07May2018 – 03Jun2018 |
| 4 | 04Jun2018 – 01Jul2018 | 5 | 02Jul2018 – 29Jul2018 | 6 | 30Jul2018 – 26Aug2018 |
| 7 | 27Aug2018 – 23Sep2018 | 8 | 24Sep2018 – 21Oct2018 | 9 | 22Oct2018 – 18Nov2018 |
| 10 | 19Nov2018 – 16Dec2018 | 11 | 17Dec2018 – 13Jan2019 | 12 | 14Jan2019 – 10Feb2019 |
| 13 | 11Feb2019 – 10Mar2019 | 14 | 11Mar2019 – 07Apr2019 | 15 | 08Apr2019 – 05May2019 |
| 16 | 06May2019 – 02Jun2019 | 17 | 03Jun2019 – 30Jun2019 | 18 | 01Jul2019 – 28Jul2019 |
| 19 | 29Jul2019 – 25Aug2019 | 20 | 26Aug2019 – 22Sep2019 | 21 | 23Sep2019 – 20Oct2019 |
| 22 | 21Oct2019 – 17Nov2019 | 23 | 18Nov2019 – 15Dec2019 | 24 | 16Dec2019 – 12Jan2020 |
| 25 | 13Jan2020 – 09Feb2020 | 26 | 10Feb2020 – 08Mar2020 | 27 | 09Mar2020 – 05Apr2020 |
| 28 | 06Apr2020 – 03May2020 | 29 | 04May2020 – 31May2020 | 30 | 01Jun2020 – 28Jun2020 |
| 31 | 29Jun2020 – 26Jul2020 | 32 | 27Jul2020 – 23Aug2020 | 33 | 24Aug2020 – 20Sep2020 |
| 34 | 21Sep2020 – 18Oct2020 | 35 | 19Oct2020 – 15Nov2020 | 36 | 16Nov2020 – 13Dec2020 |
| 37 | 14Dec2020 – 10Jan2021 | 38 | 11Jan2021 – 07Feb2021 | 39 | 08Feb2021 – 07Mar2021 |
